# Supplementary figures and images for: Investigating the use of physics informed neural networks for dam-break scenarios
Source: PLoS One. 2025 Sep 19;20(9):e0332694. doi: 10.1371/journal.pone.0332694 (PMC12449020; doi:10.1371/journal.pone.0332694)

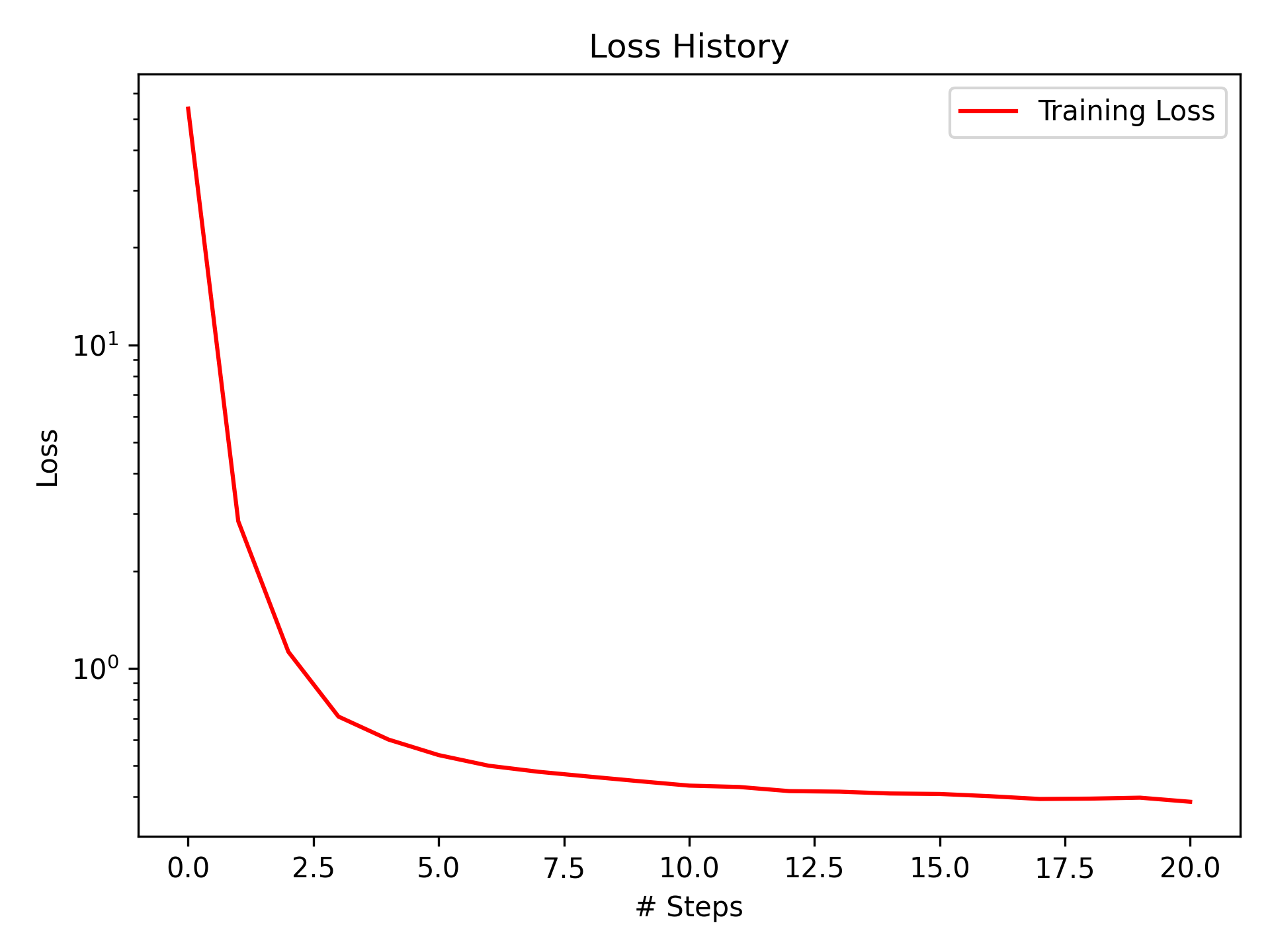

Supplement: S1 File — Dataset for the 1D and 2D shallow water equations (SWE). Repository: https://github.com/kinzamumtaz/2D_SWE. (ZIP) [file pone.0332694.s001.zip › 2D_SWE-main/Downloads/solution_outputs_2dstep/training_loss_plot_2dstep.png]

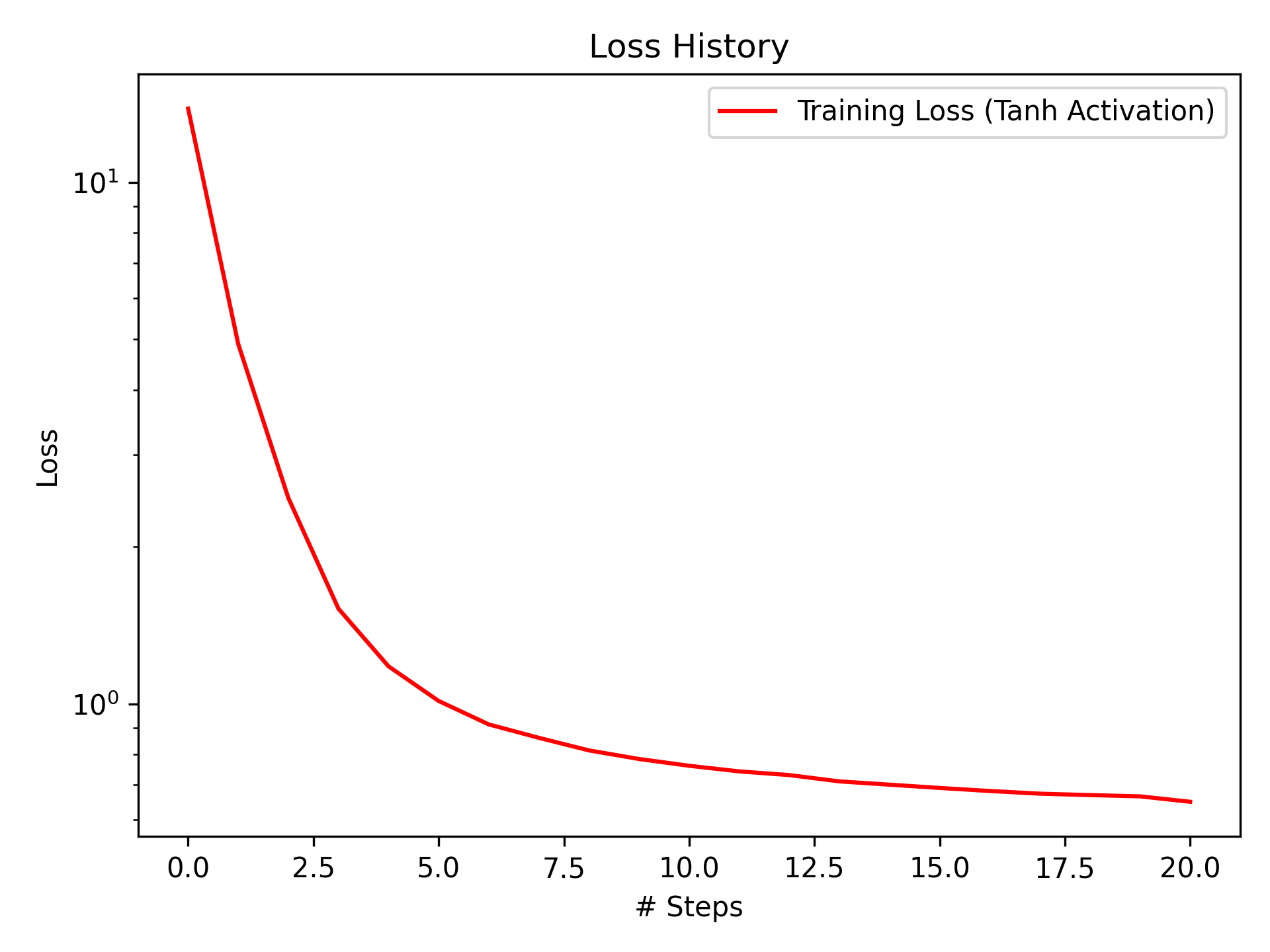

Supplement: S1 File — Dataset for the 1D and 2D shallow water equations (SWE). Repository: https://github.com/kinzamumtaz/2D_SWE. (ZIP) [file pone.0332694.s001.zip › 2D_SWE-main/Downloads/solution_outputs_circular/training_loss_circular.png]

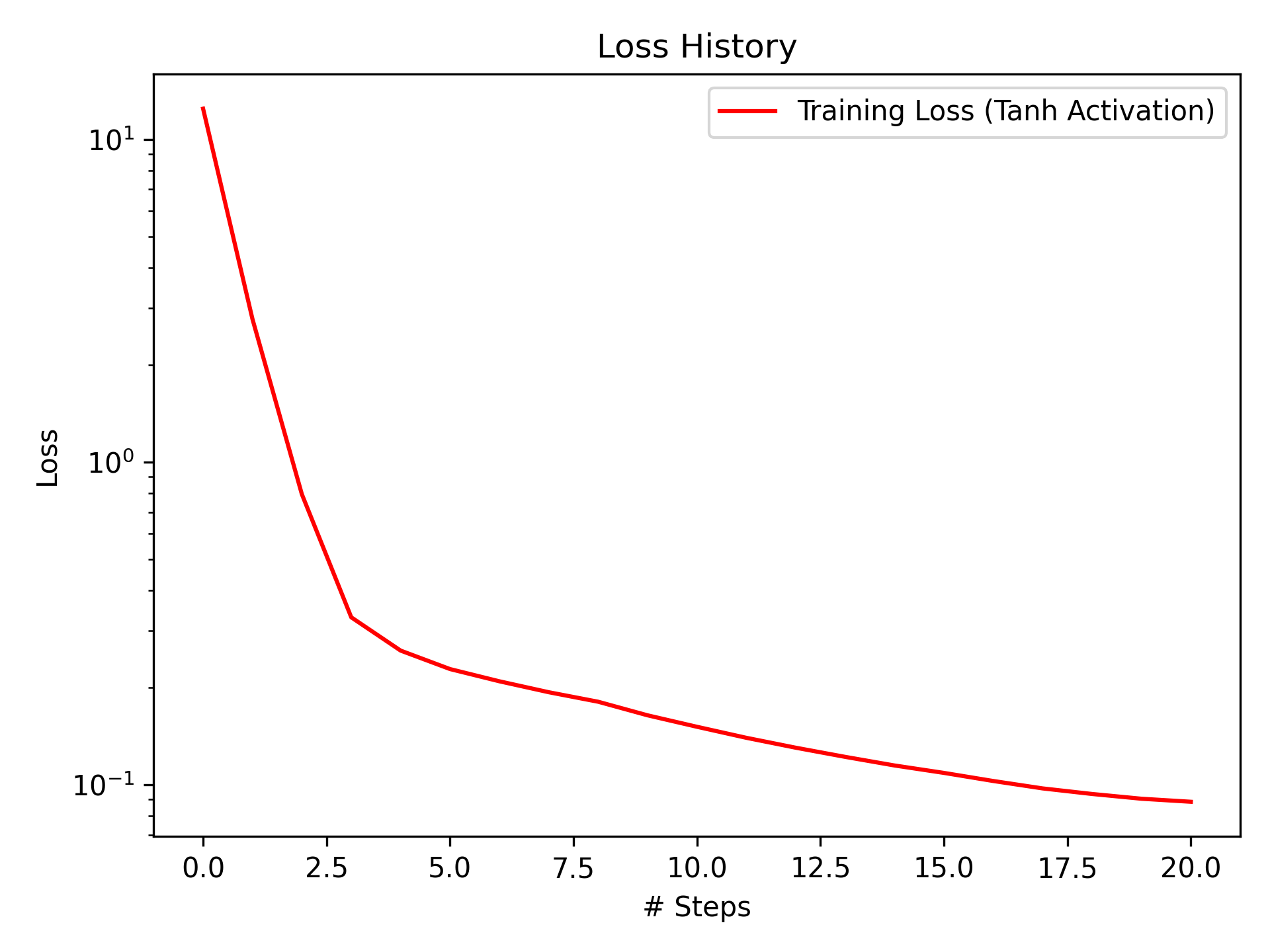

Supplement: S1 File — Dataset for the 1D and 2D shallow water equations (SWE). Repository: https://github.com/kinzamumtaz/2D_SWE. (ZIP) [file pone.0332694.s001.zip › 2D_SWE-main/Downloads/solution_outputs_circularNum/training_loss_circularNum.png]

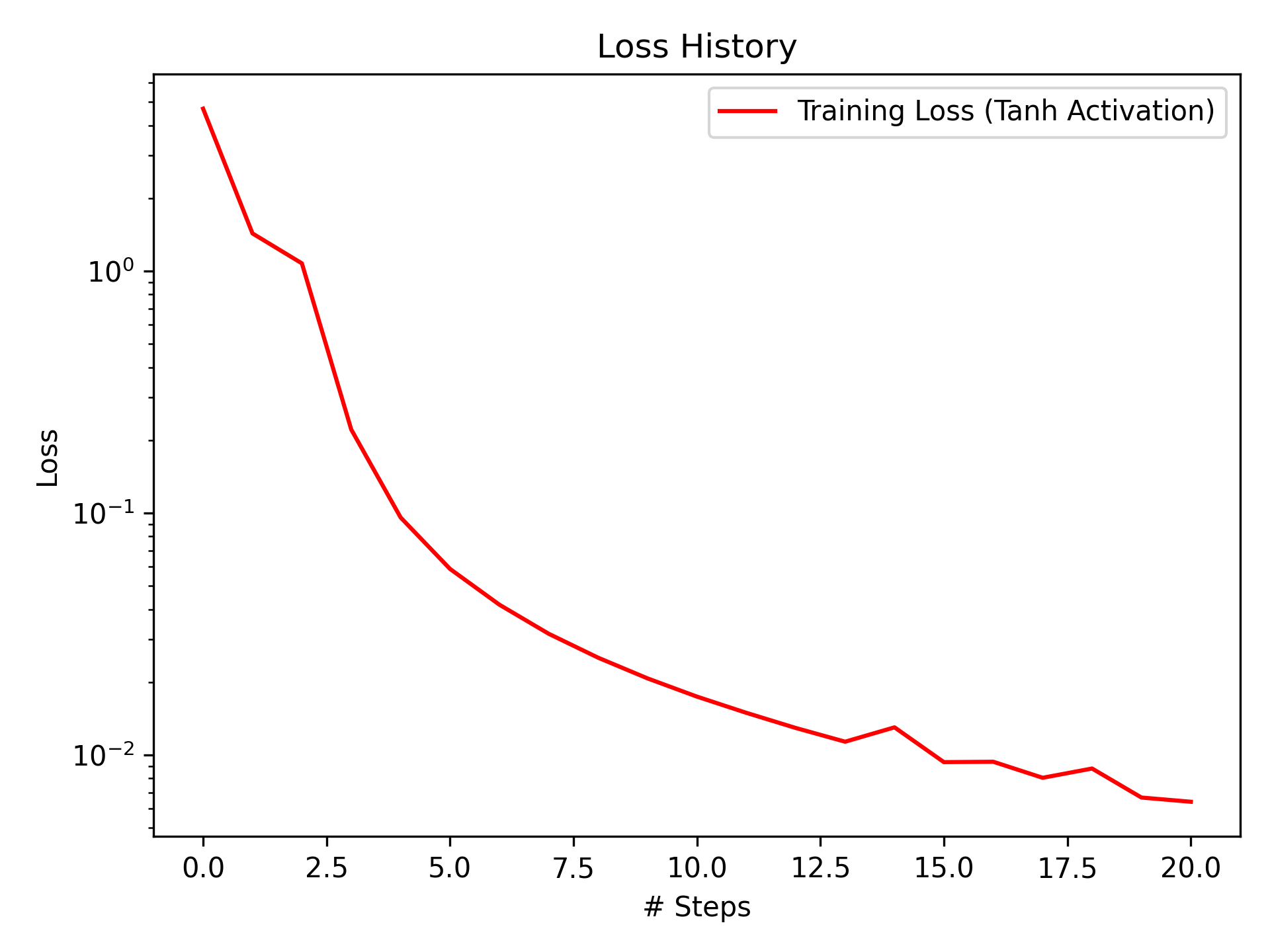

Supplement: S1 File — Dataset for the 1D and 2D shallow water equations (SWE). Repository: https://github.com/kinzamumtaz/2D_SWE. (ZIP) [file pone.0332694.s001.zip › 2D_SWE-main/Downloads/solution_outputs_gaussian/training_loss_gaussian.png]

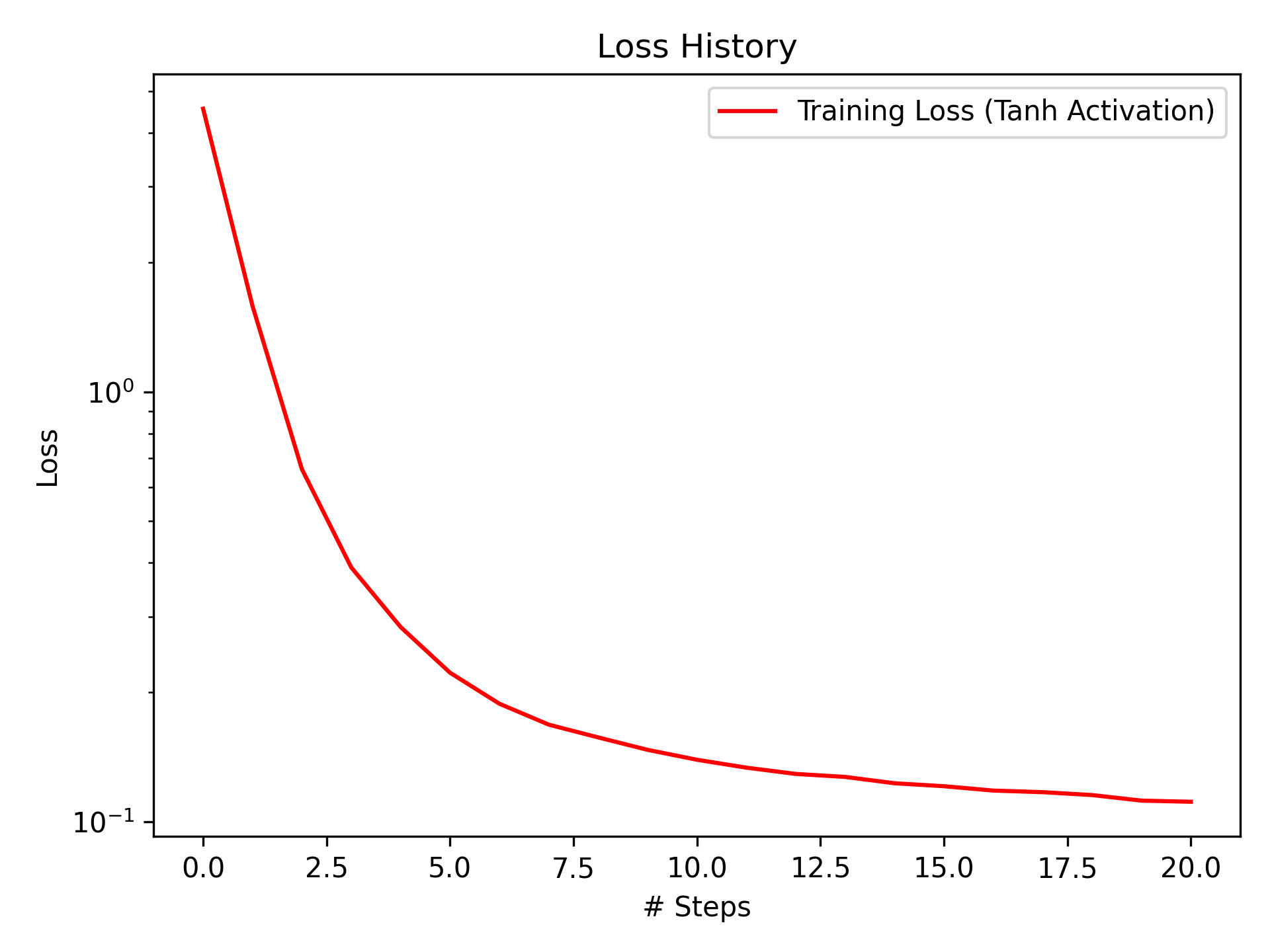

Supplement: S1 File — Dataset for the 1D and 2D shallow water equations (SWE). Repository: https://github.com/kinzamumtaz/2D_SWE. (ZIP) [file pone.0332694.s001.zip › 2D_SWE-main/Downloads/solution_outputs_rec/training_loss_rec.png]
