# Supplementary material for: Investigating the use of physics informed neural networks for dam-break scenarios
Source: PLoS One. 2025 Sep 19;20(9):e0332694. doi: 10.1371/journal.pone.0332694 (PMC12449020; doi:10.1371/journal.pone.0332694)
Supplement: Attachment — Submitted filename: Review_PlosOne.docx [file pone.0332694.s002.docx]

**Comments**

1. In the introduction section, the sentence beginning with *"In recent years, … of hydrological applications"* lacks clarity. Please rephrase it to convey a more precise meaning.
2. The manuscript currently cites only a few references ([1], [2], [3]) and lacks an in-depth comparison of related work involving PINNs in hydrology or fluid mechanics. A more comprehensive literature review is needed to establish the novelty and context of your work.
3. The manuscript contains several repetitive sentences, especially in the introduction and methodology sections. Consider revising for conciseness and clarity.
4. In Section 1.1 (*State of the Art*), phrases like *"[4] is employed..."* should be rewritten using author names, e.g., *"Kader et al., 2020 [4]."* Please ensure that all references throughout the manuscript follow this format for clarity and readability.
5. Figures 7, 9, 11, 13, and 15 are labeled as showing both training and testing loss, but only one curve is plotted, which appears to be the training loss. Moreover, these figures are not cited or discussed in the main text. Please correct the figures and provide a proper interpretation in the results section.
6. The purpose and content of Figures 4 and 6 are unclear. Please elaborate on what these figures represent and how they contribute to the findings.
7. The manuscript does not explain how the models were trained. What portions of the data were used for training and testing? What were the input features and target variables? This information is critical and must be included.
8. There is no discussion of how model performance was validated. Please include quantitative evaluation metrics such as RMSE, MAE, MSE, or R² to assess and compare the model predictions.
9. The results section lacks detailed explanation and interpretation of the figures. Each figure should be properly described and analyzed to highlight key insights and outcomes.
10. A discussion section is completely missing from the manuscript. Please add a dedicated section that critically interprets the findings, compares them with previous studies, discusses limitations, and highlights the implications of your results.
